# Fatty Acids, Lipid Mediators, and T-Cell Function

**DOI:** 10.3389/fimmu.2014.00483

**Published:** 2014-10-13

**Authors:** Anja J. de Jong, Margreet Kloppenburg, René E. M. Toes, Andreea Ioan-Facsinay

**Affiliations:** ^1^Department of Rheumatology, Leiden University Medical Centre, Leiden, Netherlands

**Keywords:** T-cells, fatty acids, lipid mediators, obesity, inflammation

## Abstract

Research toward the mechanisms underlying obesity-linked complications has intensified during the last years. As a consequence, it has become clear that metabolism and immunity are intimately linked. Free fatty acids and other lipids acquired in excess by current feeding patterns have been proposed to mediate this link due to their immune modulatory capacity. The functional differences between saturated and unsaturated fatty acids, in combination with their dietary intake are believed to modulate the outcome of immune responses. Moreover, unsaturated fatty acids can be oxidized in a tightly regulated and specific manner to generate either potent pro-inflammatory or pro-resolving lipid mediators. These oxidative derivatives of fatty acids have received detailed attention during the last years, as they have proven to have strong immune modulatory capacity, even in pM ranges. Both fatty acids and oxidized fatty acids have been studied especially in relation to macrophage and T-cells functions. In this review, we propose to focus on the effect of fatty acids and their oxidative derivatives on T-cells, as it is an active area of research during the past 5 years. The effect of fatty acids and their derivatives on activation and proliferation of T-cells, as well as the delicate balance between stimulation and lipotoxicity will be discussed. Moreover, the receptors involved in the interaction between free fatty acids and their derivatives with T-cells will be summarized. Finally, the mechanisms involved in modulation of T-cells by fatty acids will be addressed, including cellular signaling and metabolism of T-cells. The *in vitro* results will be placed in context of *in vivo* studies both in humans and mice. In this review, we summarize the latest findings on the immune modulatory function of lipids on T-cells and will point out novel directions for future research.

## Introduction

Obesity is increasing in the Western society, and obesity-linked complications are under intense scrutiny. Among these, not only metabolic disorders, such as diabetes mellitus and dyslipidemia, but also cardiovascular disorders, such as hypertension and ischemic heart diseases, have been shown to be associated with obesity (1, 2). More recently, also chronic diseases in which inflammation plays a role such as osteoarthritis, rheumatoid arthritis, inflammatory bowel disease, chronic obstructive pulmonary disease, and asthma have been associated with obesity (3–6). Likewise, evidence exists indicating a relationship between obesity and increased susceptibility for infections (7), as well as a lower response to vaccination (8) indicating that obesity can impact immune responses. Although the mechanisms underlying these associations are unclear, adipose tissue-derived inflammation could play a role in the development and progression of these diseases. Indeed, adipose tissue serves not only as an energy depot but it is also a highly active metabolic and endocrine organ, affecting whole body metabolism (3, 9, 10).

The adipose tissue consists of adipocytes and the stromal vascular fraction, in which a variety of immune cells can be found. Among these, macrophages and T-cells are the most abundant (11). Expansion of the adipose tissue is accompanied by an increased infiltration of immune cells with a pro-inflammatory phenotype. The cross-talk between the infiltrating cells and the tissue-resident adipocytes leads to secretion of adipokines, cytokines, chemokines, and lipids with a predominant pro-inflammatory character (3, 10). Moreover, the levels of various adipokines and cytokines are altered in obese individuals compared to lean ones (e.g., leptin, adiponectin, IL-6) (12).

This cross-talk has also been shown to affect the function of adipocytes, such as lipolysis, which will most likely result in an altered concentration of circulating free fatty acids. Indeed, obese persons have higher levels of free fatty acids in plasma compared to lean persons (13–15). Whether and which of these soluble factors (adipokines, cytokines, lipids, etc.) contribute to obesity-mediated inflammatory effects in diseases is still under investigation.

Several indirect lines of evidence suggest that fatty acids can modulate the immune response. One of these is that levels of several fatty acids are associated with levels of inflammatory markers in healthy individuals (16). More directly, the type of fatty acids contained in the diet has been suggested to influence the risk of development of inflammatory diseases in which the immune system plays an important role. Fatty acids from animal sources, such as meat and poultry, are mainly saturated fatty acids or omega-6 (ω6) unsaturated fatty acids, while fatty acids derived from plant-based foods, oils, and certain types of fatty fish consist mainly of ω3 unsaturated fatty acids. Diets rich in ω6 polyunsaturated fatty acids (PUFAs) increase the risk of development of inflammatory diseases, such as rheumatoid arthritis, inflammatory bowel disease, and asthma (17). On the other hand, diets rich in ω3 PUFAs seem to have anti-inflammatory effects as indicated both by the decreased risk and the amelioration of these diseases (17–20). Moreover, it has been shown that after *in vivo* challenge with a pathogen, the host survival and pathogen clearance is affected by diets enriched in fatty acids, although this is dependent on the infectious agent and the fatty acids used (21, 22).

Additionally, unsaturated fatty acids can be oxidized to generate either potent pro-inflammatory or pro-resolving lipid mediators. These lipid mediators have strong immune modulatory capacity and are generated in a timely and controlled manner during an inflammatory response (23). Pro-inflammatory lipid mediators, such as prostaglandins and leukotrienes, are produced first. When the tissue-damaging or infectious agent is removed, the production of pro-resolving lipid mediators associates with restoration of normal tissue homeostasis (24).

In conclusion, several publications indicate that fatty acids and lipid mediators derived from fatty acids can potently influence the immune system. Although it is often unclear which cells are responsible for the immune modulatory effects of fatty acids, these lipids have been shown to affect the function of several immune cells, especially antigen-presenting cells and T-cells (25, 26). In this review, we propose to summarize the knowledge regarding the effect of fatty acids and their oxidative derivatives on T-cells, both *in vitro* and *in vivo*, as it has been an active area of research during recent years.

## Fatty Acids

Fatty acids serve not only as fuel for cells but are also components of cell membrane phospholipids and glycolipids, and precursors of bioactive lipid mediators (27). Fatty acids are hydrocarbon structures, which can differ in the number of carbon atoms and the degree of saturation. Therefore, they are classified according to length (number of carbon residues in the lipid backbone), saturation, and number and position of the double bonds. Fatty acids can be either short-chain (4–10 carbons), medium-chain (12–14 carbons), long-chain (16–18 carbons) or very long-chain fatty acids (20 or more carbons). In addition, these lipids can be classified according to the presence or absence of a double bond in the backbone. Fatty acids that do not have a double bond are called saturated fatty acids and those having one or more double bonds are called monounsaturated fatty acids (MUFAs) or PUFAs, respectively. The position of the double bond determines whether PUFAs can be characterized as ω3, ω6, or ω9, whereby ω indicates the methyl end of the fatty acid and the number indicates the carbon counted from the methyl end that contains a double bond. Table 1 shows an overview of fatty acids most commonly used in literature. Some of the PUFAs, especially AA, DHA, and EPA and their influence on the immune system have been extensively reviewed elsewhere (27, 28), therefore, they will be excluded from the present review.

**Table 1:** **Examples of saturated, monounsaturated, and polyunsaturated fatty acids that are most commonly used in literature**.

Type	Isomer	Name
Saturated	4:0	Butyric acid
	12:0	Lauric acid
	14:0	Myristic acid
	16:0	Palmitic acid (PA)
	18:0	Stearic acid (SA)
Monounsaturated	18:1 (ω9)	Oleic acid (OA)
Polyunsaturated	18:2 (ω6)	Linoleic acid (LA)
	18:3 (ω3)	Alpha-linolenic acid (ALA)
	18:3 (ω6)	Gamma-linolenic acid (GLA)
	20:4 (ω6)	Arachidonic acid (AA)
	20:5 (ω3)	Eicosapentaenoic acid (EPA)
	22:5 (ω3)	Docosapentaenoic acid (DPA)
	22:6 (ω3)	Docosahexaenoic acid (DHA)

## Effect of Fatty Acids on T-Cells

Several studies have focused on the effect of fatty acids on T-cells. Although these studies differ in experimental setting, especially regarding the concentration of the fatty used, the type of T-cell studied, and the T-cell stimulus, the overall data suggest that low concentrations of fatty acids can influence the proliferation of T-cells, whereas higher concentrations induce apoptosis in a dose-dependent manner, through the induction of several pathways (29–34). In addition, the concentration at which apoptosis occurs is also determined by the degree of saturation and the length of the fatty acid. While short-chain fatty acids (SCFA) are not toxic even at a concentration of 800 μM or higher (30), longer, and more unsaturated fatty acids can already be toxic in low concentrations [e.g., linoleic acid (LA)] is toxic at 100 μM (30–32).

In contrast to the apoptotic effects, the modulatory effects of low, non-toxic concentrations of fatty acids on proliferation do not seem to correlate with length of the fatty acids tested. There are only a few studies studying the proliferation of T-cells after incubation with fatty acids and they use different stimuli (29, 33, 35). While proliferation of anti-CD3/anti-CD4 stimulated T-cells was not influenced by palmitic acid (PA) (29), concanavalin A (ConA) stimulated T-cells proliferated less in the presence of PA (33), indicating that fatty acids can have different effects depending on the stimulus used for T-cell activation. This is supported by the fact that while proliferation of T-cells stimulated with ConA was differentially affected by the saturated fatty acids stearic acid (SA), and the unsaturated fatty acids oleic acid (OA) and LA (33), stimulation with anti-CD3/anti-CD28 led to increased proliferation with all tested fatty acids [e.g., LA, SA, γ-linoleic acid (GLA), PA, OA, and palmitoleic acid] independently of saturation degree (35). Overall, the data indicate that different fatty acids can modulate proliferation of T-cells, although this is likely dependent on the stimuli used.

Only a handful of studies have investigated cytokine production by T-cells treated with fatty acids and they generally indicate that fatty acids can modulate secretion of several cytokines, such as TNFα, IL-6, IL8, IL1β, IL-2, IL10, and IFNγ (36–38). This modulation has been shown primarily for unsaturated fatty acids such as OA and LA using T-cells activated by a polyclonal stimulus, such as ionomycin (38) or ConA (37). The effect of saturated fatty acids on cytokine secretion by activated T-cells is unclear. However, saturated fatty acids induced cytokine secretion in T-cells in the absence of T-cell activation in a dose-dependent manner (36), while unsaturated fatty acids were unable to do so.

In conclusion, fatty acids are toxic to T-cells when administered in high concentrations and can induce proliferation and cytokine production when administered in non-toxic concentrations. The effects of fatty acids on T-cell function seem to be dependent not only on the saturation grade and length of fatty acids but also on the T-cell stimulus used.

## Mechanisms by Which Fatty Acid Exert Their Effects

How fatty acids exert their effects on different immune cells is incompletely understood, despite the fact that several possible mechanisms have been proposed. One possibility is that fatty acids can diffuse through the membrane of T-cells in a passive process, as some studies have shown that T-cells incorporate fatty acids in their membrane upon exposure (38, 39).

In addition to this passive mechanism, there are several active pathways possible, in general mediated by cellular receptors. Among the receptors involved in the recognition of free fatty acids, fatty acid transport proteins (FATPs) form a family of six transmembrane proteins, with distinct tissue expression patterns (40). FATPs are generally involved in the uptake of fatty acids, although there are no studies regarding their expression in T-cells. In addition to FATP, the fatty acid binding proteins (FABPs) are a family of homologous cytoplasmic proteins with high affinity for fatty acids. Even though FABPs are cytoplasmic proteins, it has been suggested that these FABPs play a role in uptake, transport, storage, and metabolism of fatty acids, with distinct patterns of tissue expression (41, 42). Interestingly, one of these receptors, the epidermal-FABP (FABP5) is also expressed on CD4^+^ T-cells (43).

Furthermore, a series of G protein-coupled receptors have been recently identified, which recognize fatty acids with different affinities and expression patterns. Five different receptors have been identified that recognize extracellular fatty acids with different lengths: free fatty acid receptor 1 (FFA1; GPR40) and GPR120 recognize long-chain fatty acids, GPR84 recognizes medium-chain fatty acids, while SCFA are recognized by FFA2 (GPR43) and FFA3 (GPR41) (44–46). The expression pattern of these receptors on T-cells has been only partly investigated. These studies indicated that FFA1 and FFA3 are not expressed on T-cells (47, 48), while the medium-chain fatty acid receptor, GPR84, is expressed by CD4^+^ and CD8^+^ cells although less abundantly than by monocytes and neutrophils (49). Smith et al. recently showed that FFA2 is expressed on colonic regulatory T-cells (cTreg) (50).

In conclusion, several mechanisms could be involved in the modulation of T-cells function by fatty acids; a passive mechanism, in which the fatty acids diffuse through the membrane or active mechanisms, in which FATPs, FABPs, or other receptors could be involved. Their relative contribution to fatty acids uptake awaits further investigation.

## Downstream Effects of Fatty Acids

There are several studies regarding downstream effects of fatty acids. These studies focus mainly on the effects of OA, LA, or PA on resting human T-cells (36) or Jurkat cells (T-cell leukemia cell line) (31, 34, 37, 51, 52). As previously mentioned fatty acids can influence T-cells in a dose-dependent manner, with low doses modulating proliferation and higher doses inducing apoptosis. Treatment of T-cells with fatty acids induces neutral lipid accumulation, such as triacylglycerol and cholesterol esters, and incorporation of the fatty acids in phospholipids (31, 34, 37). The induction of neutral lipid accumulation is mediated by activation of insulin signaling pathways and glucose utilization, indicated by elevated expression of insulin receptor and GLUT4 and by increased glucose consumption and lactate production (34, 36).

It is believed that this pathway enables T-cells to evade the toxic effects of fatty acids at both low and high concentrations. However, when T-cells are exposed to too high concentrations, these mechanisms might not be sufficient to protect the cells from apoptosis and these results in the induction of intrinsic apoptotic pathways (31, 34, 37).

Several apoptotic characteristics are induced by fatty acids in T-cells, such as mitochondrial depolarization (31, 34), caspase activation (34, 37), DNA fragmentation (31, 34), chromatin condensation (31), cytochrome *c* (34), and phosphatidylserine externalization (31), indicating that T-cells treated with high-concentrations fatty acids die due to apoptosis. In addition, T-cells incubated with fatty acids also induce oxidative and nitrosative stress leading to apoptosis, indicated by higher levels of reactive oxygen species (ROS), reactive nitrogen species, and lower levels of catalase activity (34, 51, 52).

How these mechanisms would affect proliferation and cytokine production of T-cells is unclear and awaits further investigation. Likewise, it is unclear whether these mechanisms are still utilized by T-cells that have been activated by their cognate antigen on antigen-presenting cells and which display an increased energy demand in order to proliferate, differentiate, and execute their effector functions.

**Figure 1:**
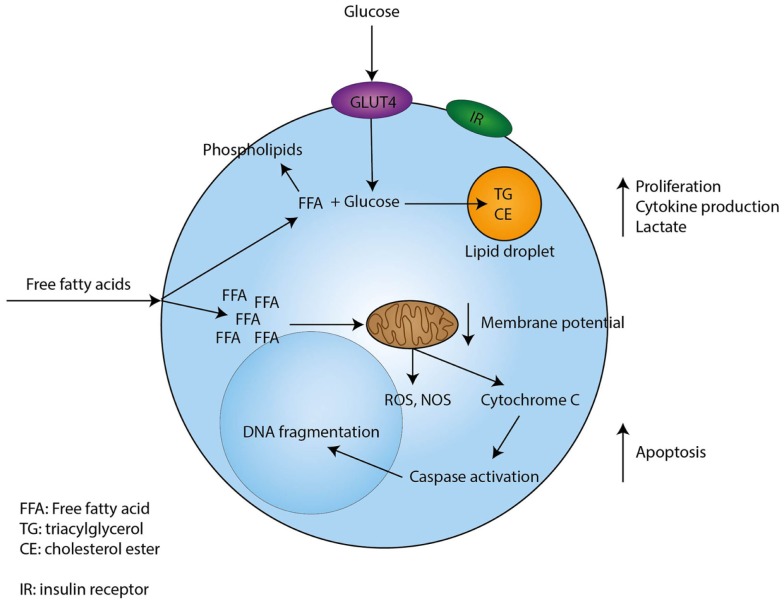
**Proposed mechanism through which free fatty acids exert their effects on T-cells**. Free fatty acids enter T-cells through currently unknown mechanisms. Low concentrations of free fatty acids are incorporated in phospholipids, while uptake of glucose sustains the formation of triacylglycerol and cholesterol esters. In addition, low concentrations of free fatty acids induce proliferation, cytokine production, and lactate production by T-cells. However, when a T-cell encounters high concentrations of free fatty acids this will lead to depolarization of the mitochondrial membrane and the induction of intrinsic apoptotic pathways, which eventually lead to apoptosis.

In conclusion, free fatty acids induce proliferation of resting T-cells in low concentrations, while higher concentrations induce apoptosis. In Figure 1, we present possible mechanisms involved in the modulatory effects of fatty acids in T-cells.

## *In vivo* Relevance

As mentioned above, obese people express generally higher plasma levels of free fatty acids after fasting when compared to healthy controls. Likewise, the turnover rate of free fatty acids is higher after fasting (14, 15). Furthermore, some studies indicated T-cell numbers are decreased in obese persons (53–55) and T-cell proliferation and T-cell subset composition are altered compared to lean persons (54–57). The mechanisms underlying these differences between obese and lean individuals are unclear. However, some studies addressing the effect of dietary fatty acid content on the function of T-cells, both in rodent models (58–62) and in humans (63, 64) suggest that fatty acids can be modulators of T-cell function and phenotype also *in vivo*.

Rodent studies show that different high-fat diets can decrease PHA (58) and conA (60) induced proliferation of T-cells when compared to control diets. Furthermore, rodent models showed that diets rich in fatty acids can have an effect on IL-2 production and signaling, since IL-2 production (58) and the expression of IL-2 receptor α-chain (CD25) (59) were increased when mice or rats are fed fatty acid rich diets. More recently, some studies investigated the effects of SCFAs on the immune system. SCFAs are metabolites of microbiota in the gut and are believed to play an important role in the balance between pro-inflammatory and anti-inflammatory effects in the gut. Indeed, recent studies show that adding SCFAs, especially butyrate and propionate, to the diet leads to induction of T-regulatory cells in the gut (61, 62). In line with this, butyrate was shown to ameliorate T-cell-dependent experimental colitis (61). In summary, these studies indicate that fatty acids can affect T-cell proliferation, cytokine production, and skewing *in vivo*.

There are only two studies regarding the effect of dietary change of fatty acid content on the function of T-cells in humans (63, 64). A diet containing MUFAs did not affect conA stimulated T-cell proliferation (63), while another study using several diets with different fatty acids composition described a higher PHA and conA induced proliferation of T-cells compared to baseline for most diets investigated (64). A direct comparison of these studies is, however, difficult due to differences in diet composition.

Overall, the data suggest that fatty acids can influence both rodent and human T-cells *in vivo*. A firm conclusion is, however, difficult to formulate, as rodent studies show decrease in proliferation, while human studies either show unaffected proliferation or induction of proliferation. Furthermore, it remains uncertain whether the effects of saturated and unsaturated fatty acids are different.

## Lipid Mediators

Lipid mediators are produced through conserved biosynthetic pathways involving specific enzymes, which exert their function on lipid precursors that are released from membranes. There are several families of lipid mediators, which can be divided into pro-inflammatory lipid mediators and the more recently described specialized pro-resolving lipid mediators (SPM). Pro-inflammatory lipid mediators include prostaglandins and leukotrienes, while SPM include lipoxins, resolvins, maresins, and protectins (23).

## Pro-Inflammatory Lipid Mediators and T-Cells

Prostaglandins and their influence on T-cells are widely studied and these studies have been reviewed elsewhere (65, 66). In short, PGE2 is the most studied prostaglandin and it has been shown to impair proliferative responses of T-cells (67–69). In addition, PGE2 can affect T-cell cytokine production, whereby Th17 associated cytokines, such as IL17, are upregulated (70–73). Moreover, PGE2 can change the Th1/Th2 balance by favoring Th2 skewing *in vitro* and in a mice model (69, 74). The effect of PGE2 on skewing of naïve T-cells toward Th17 cells is uncertain since there are contradicting studies showing either an induction of Th17 cells (75) or a reduction of Th17 cells (76), depending on the combination of cytokines used to induce Th17 differentiation. PGE2 can exert these effects on T-cells through E-prostanoid (EP) 2 receptor and EP4, which are present on T-cells (72, 73, 75). So, prostaglandins can affect T-cell proliferation and T-cell skewing through the EP2 and EP4 receptors.

Another family of pro-inflammatory lipid mediators are the leukotrienes, but the effects of leukotrienes on T-cells are not completely known. A human study revealed that both Th1 and Th2 express cysteinyl leukotriene receptor 1 (CYSLTR1) mRNA, although expression of CYSLTR1 was higher in Th2 cells compared to Th1 cells. In addition, activation of the CYSTLR1 with LTD4, LTC4, or LTE4 led to the induction of calcium signaling in Th2 cells, but a much weaker response in Th1 cells. LTD4 can also act as chemo attractant for Th2 cells (77). This indicates that Th2 cells are more susceptible than Th1 cells to respond to leukotrienes.

Different *in vivo* mice models have indicated that mice deficient in 5-lipoxygenase (5-LO), which is one of the key enzymes in leukotriene production, have less T-cells infiltrating the inflamed area (78–81), diminished numbers of CD4^+^CD25^+^ regulatory T-cells, higher levels of IFNγ, and increased T-bet (Th1 lineage commitment) (80). In addition, LTB4 dose dependently down-regulates the differentiation of naïve T-cells into Tregs, while it enhances the differentiation of Th17 cells, with more IL17 secretion and higher levels of RORγt mRNA expression (82).

In summary, pro-inflammatory lipid mediators seem to stimulate T-cell migration (either directly or indirectly) and to favor skewing toward Th2 or Th17 instead of Th1 or Tregs.

## Pro-Resolving Lipid Mediators and T-Cells

Pro-resolving lipid mediators were recently described and have been shown to be involved in the resolution phase of inflammation. There are only a few studies investigating the effects of pro-resolving lipid mediators on T-cells; however, these studies indicate that these lipid mediators can influence T-cells.

Lipoxins, especially lipoxin A4 (LXA4) and lipoxin B4 (LXB4), have been shown to inhibit TNFα secretion from both PBMCs and peripheral T-cells stimulated with anti-CD3 Abs (83), and the effects observed with LXA4 were mediated by the LXA4 receptor (FPR2/ALX), expressed on T-cells (83, 84). FPR2/ALX expression is higher on activated CD25^+^ and memory CD45RO^+^ CD4^+^ T-cells when compared to CD25^−^ and naïve CD45RA^+^ CD4^+^ T-cells (84), indicating that activated T-cells can respond better to the agonist of this receptor.

Administration of resolvin D1 (RvD1) led to reduced infiltration of CD4^+^ and CD8^+^ T-cells in the perivascular tissue of the eye of LPS induced uveitis in rats, resulting in the amelioration of the disease. This suggests that RvD1 prevents migration of T-cells into the eye, either directly or indirectly, thereby preventing disease in a rat model (85).

Moreover, protectin 1 (PD1) could reduce T-cell infiltration in zymosan A induced peritonitis (86) and into the lungs of OVA-sensitized and challenged mice (87). Furthermore, *in vitro* studies show that PD1 inhibits cytokine secretion, such as TNFα and IFNγ by T-cells and can induce apoptosis of T-cells (86). Therefore, PD1 could influence the inflammatory response by preventing the migration of T-cells and induction of apoptosis of T-cells.

In contrast to their pro-inflammatory counterparts, pro-resolving lipid mediators appear to inhibit cytokine production and migration of T-cells and to induce their apoptosis, thereby promoting resolution of inflammation.

## Future Research Plans

In this review, we have summarized the current knowledge on the influence of fatty acids and lipid mediators on T-cells. These data indicate that fatty acids and their derivatives do affect the function and phenotype of T-cells and that the type and concentration of free fatty acids determines the outcome of this modulation and most likely the outcome of T-cell-dependent immune reactions. However, the presented data indicate that substantial further research is needed as there are still several remaining questions that need to be elucidated. These include a more detailed insight into the mechanisms involved in fatty acid uptake and usage by the cells, as well as into the possible *in vivo* impact of these changes.

Although some data exist on the effect of fatty acids on T-cell proliferation and cytokine production, these are inconsistent due to usage of different stimuli for T-cells. Future research should probably focus on more physiological stimuli, including antigen-specific stimulation using antigen-presenting cells. Moreover, the molecular events linking fatty acid uptake to modification of T-cell function and phenotype need to be identified, as they could constitute drug targets for future therapies of obesity-linked complications. Likewise, it is of interest whether stimulated cells use the same mechanisms as unstimulated cells to evade the toxic effects and the induction of proliferation and cytokines. Stimulated cells have a different energy demand than unstimulated cells, and therefore, could respond differently to fatty acids. This would be directly relevant for understanding modulation of ongoing immune responses by free fatty acids and consequently for development of efficient vaccination strategies or efficient drugs in T-cell-mediated diseases.

Additionally, *in vivo* studies systematically investigating the effect of various dietary fatty acids on T-cells would be needed as the current data are scarce and inconsistent. The latter is most likely due to a lack of standardization in the diets and the outcome measures used in the published studies. Also, current research is mainly focused on ω3 and ω6-rich diets, while relatively little is known about other fatty acids, which are also abundant in diet, such as OA, palmitic and palmitoleic acid, and others. Future research about the effect of these fatty acids is needed.

Current knowledge regarding the effects of lipid mediators on T-cells is mainly focused on pro-inflammatory lipid mediators. Therefore, research both *in vitro* and *in vivo* addressing the effects of pro-resolving lipid mediators on T-cell proliferation, differentiation, and function would be of interest. This would not only provide novel insights into the link between inflammation resolution and the adaptive immunity, which is a yet unexplored area of research, but could also lead to identification of new drug targets for modulation of T-cell responses.

## Concluding Remarks

The Western world experiences an epidemic of obesity due to excess intake of food. Among the many nutrients acquired in excess, free fatty acids have an important impact on various bodily functions, as recent research has made clear. In this review, we have provided an overview of the current knowledge about the effects of fatty acids and their derivatives on T-cells. The summarized data indicate that the effect of fatty acids on T-cells is either stimulatory or lipotoxic, depending on the capacity of the T-cells to evade the toxic effects of the fatty acids. Both fatty acids and their oxidative derivatives can influence T-cell proliferation, skewing, and differentiation and this is usually dependent on the type of fatty acid. This suggests that the type of fatty acid in the diet, rather than its quantity, determine the outcome of an immune reaction in a certain individual. Future research will teach us whether and how fatty acids or lipid mediators can be used as drug targets in treating obesity-related or T-cell-driven diseases.

## Conflict of Interest Statement

The authors declare that the research was conducted in the absence of any commercial or financial relationships that could be construed as a potential conflict of interest.
